# Periodontitis as a potential amplifier of diabetes-related genitourinary complications: evidence gradients and mechanistic insights into the inflammation–microvascular injury axis

**DOI:** 10.3389/fcimb.2026.1843576

**Published:** 2026-06-10

**Authors:** Zhiyuan Bai, Xiaoming Lin, Binbin Wang, Zhanhao Li, Xiaoguang Qu, Yuchuan Hou

**Affiliations:** 1 Department of Urology I, The First Hospital of Jilin University, Changchun, China; 2 Department of Gastric and Colorectal Surgery, General Surgery Center, The First Hospital of Jilin University, Changchun, China; 3 Department of Urology II, The First Hospital of Jilin University, Changchun, China; 4 Urology Department, Jilin Central General Hospital, Jilin City, China

**Keywords:** diabetes mellitus, genitourinary complications, inflammation, microvascular injury, periodontitis

## Abstract

Periodontitis is increasingly recognized as a chronic systemic inflammatory burden that may be associated with greater vulnerability to selected diabetes-related genitourinary complications through overlapping inflammatory and microvascular pathways. This review integrates current epidemiological, mechanistic, and clinical evidence and proposes a conceptual “oral–metabolic–genitourinary axis” to describe potential links between periodontal inflammation and diabetic kidney disease (DKD), diabetes-related erectile dysfunction (ED), and recurrent urinary tract infections (UTIs). Available evidence is strongest for renal endpoints: observational studies and recent cohort data suggest associations between periodontitis and albuminuria, renal function decline, or dialysis risk in patients with type 2 diabetes. In contrast, evidence for ED and recurrent UTIs remains limited, with much of the support derived from mechanistic inference and indirect clinical observations. The proposed biologically plausible pathways include amplification of chronic low-grade systemic inflammation, endothelial and microvascular dysfunction, oxidative stress, advanced glycation end products–receptor for advanced glycation end products (AGE–RAGE) signaling, and microbiome interactions involving the oral–gut–genitourinary axis. These proposed associations and pathways may be modified or intensified by poor glycemic control, obesity, smoking, vitamin D deficiency, and gut dysbiosis. Clinically, periodontal therapy has been associated with improved glycemic control and may improve selected inflammatory or renal-related surrogate indicators, suggesting that oral health management could be considered a supportive component of multidisciplinary diabetes care. Overall, periodontitis is best viewed at present as a plausible amplifying factor rather than a confirmed independent cause of these outcomes, and this hypothesis requires confirmation in large prospective cohorts, randomized trials, and multi-omics studies.

## 1 Introduction

Periodontitis has traditionally been regarded as a localized inflammatory disease of the supporting tissues of the teeth, but increasing evidence indicates that its biological effects may extend beyond the oral cavity. Periodontitis is driven by dysbiotic plaque biofilms and an exaggerated host immune-inflammatory response, leading to gingival inflammation, periodontal pocket formation, clinical attachment loss, and alveolar bone resorption (Abdulkareem et al., 2023). Beyond local tissue destruction, periodontal pathogens, microbial products, and inflammatory mediators may enter the systemic circulation through a disrupted gingival barrier, thereby contributing to low-grade systemic inflammation and potentially affecting distal organs (Hajishengallis and Chavakis, 2021; Vitkov et al., 2023). In patients with diabetes, chronic hyperglycemia, immune dysregulation, advanced glycation end products–receptor for advanced glycation end products (AGE–RAGE) signaling, and microvascular injury create a systemic environment in which periodontal inflammation may have broader metabolic and vascular consequences (Gurav, 2012; Preshaw et al., 2012; Yang Y. et al., 2024). This raises an important question: whether periodontitis may act as a modifiable inflammatory and microvascular amplifier of diabetes-related complications beyond the well-established bidirectional relationship between diabetes and periodontal disease (Preshaw et al., 2012; Park et al., 2022).

Among diabetes-related complications, genitourinary outcomes are clinically important but have not been discussed extensively from an oral-systemic perspective. Diabetic kidney disease (DKD) is one of the most serious microvascular complications of diabetes and remains a major cause of chronic kidney disease and end-stage renal disease worldwide (de Boer et al., 2022; Młynarska et al., 2024). Erectile dysfunction (ED) is common among men with diabetes and is closely linked to endothelial dysfunction, neuropathy, chronic inflammation, and oxidative stress (Defeudis et al., 2022; Dilixiati et al., 2024). Patients with diabetes are also more susceptible to urinary tract infections (UTIs), particularly recurrent and complicated infections, owing to hyperglycemia-associated immune dysfunction, altered urinary tract defenses, and bladder dysfunction (Nitzan et al., 2015; Geerlings, 2016; Papp and Zimmern, 2023). Although DKD, diabetes-related ED, and recurrent UTIs differ substantially in clinical presentation, they share partially overlapping biological features, although the degree of overlap differs by outcome. DKD and diabetes-related ED are both closely linked to chronic inflammation, endothelial dysfunction, oxidative stress, and microvascular injury (Defeudis et al., 2022; Dilixiati et al., 2024; Młynarska et al., 2024). Recurrent UTIs in diabetes are more closely related to hyperglycemia-associated immune dysfunction, altered urinary tract defenses, and bladder dysfunction (Nitzan et al., 2015; Geerlings, 2016; Papp and Zimmern, 2023).

The relationship between diabetes and periodontitis is widely recognized as bidirectional (Preshaw et al., 2012). Diabetes increases susceptibility to periodontitis by impairing immune responses, altering collagen metabolism, promoting AGE–RAGE signaling, and aggravating microvascular injury (Gurav, 2012; Preshaw et al., 2012; Yang Y. et al., 2024). Conversely, periodontitis has been associated with poorer glycemic control and higher systemic inflammatory burden, which may be relevant to increased vulnerability to diabetes-related complications (Gurav, 2012; Preshaw et al., 2012; Yang Y. et al., 2024). Previous population-based evidence has suggested that periodontitis is associated with microvascular complications of diabetes, supporting the biological plausibility of an oral-systemic link in diabetes-related target-organ injury (Park et al., 2022). However, the extent to which periodontitis is associated with specific genitourinary complications of diabetes remains unevenly supported across outcomes. Current evidence is strongest for renal outcomes, including albuminuria, renal function decline, and dialysis-related endpoints. By contrast, evidence linking periodontitis to diabetes-related ED is more limited and is derived largely from non-diabetic or mixed populations, whereas evidence for recurrent UTIs in patients with diabetes remains mostly indirect and mechanistic.

On this basis, the present narrative review proposes an “oral–metabolic–genitourinary axis” as a conceptual framework. This framework is used to discuss how periodontitis-related inflammation, endothelial and microvascular dysfunction, oxidative stress, AGE–RAGE signaling, and microbiome interactions may overlap with diabetes-related pathogenic pathways. This framework is not intended to imply that periodontitis is a confirmed independent cause of all diabetes-related genitourinary complications. Rather, periodontitis is best considered, based on current evidence, as a plausible and potentially modifiable amplifying factor. Accordingly, this review focuses on DKD as the most evidence-supported endpoint, while discussing diabetes-related ED and recurrent UTIs as clinically relevant but less established extensions of the same biological theme.

## 2 Review methodology

This article was designed as a narrative review based on a structured literature search. The primary aim was to integrate current knowledge on periodontitis, diabetes-related metabolic injury, systemic inflammation, microvascular dysfunction, oxidative stress, AGE–RAGE signaling, and microbiome interactions, and to discuss how these mechanisms may converge in diabetes-related genitourinary complications. Clinical and epidemiological evidence was reviewed to provide context for the proposed oral–metabolic–genitourinary axis, rather than to perform a formal quantitative comparison across outcomes.

The literature search was conducted primarily in PubMed/MEDLINE and was supplemented by searches in Web of Science, Scopus, Google Scholar, and the reference lists of relevant articles. Searches included literature published from database inception to May 2026. The following search terms were used alone and in combination: “periodontitis,” “periodontal disease,” “diabetes mellitus,” “type 2 diabetes,” “diabetic kidney disease,” “chronic kidney disease,” “albuminuria,” “renal function decline,” “erectile dysfunction,” “urinary tract infection,” “recurrent urinary tract infection,” “inflammation,” “endothelial dysfunction,” “microvascular injury,” “oxidative stress,” “AGE-RAGE,” “microbiome,” “oral-gut axis,” “oral-systemic inflammation,” “host susceptibility,” “genetic polymorphism,” “Toll-like receptor 4 (TLR4),” and “omics”.

Eligible publications included peer-reviewed original studies, including observational, cohort, interventional, and randomized controlled studies, as well as systematic reviews, meta-analyses, and relevant mechanistic studies or reviews relevant to periodontal inflammation, diabetes-related metabolic injury, endothelial and microvascular dysfunction, AGE–RAGE signaling, oxidative stress, microbiome interactions, and genitourinary outcomes. Publications were excluded if they were unrelated to periodontitis, diabetes, genitourinary complications, or the shared inflammatory, metabolic, microvascular, and microbiome-related pathways considered in this review. Unpublished data, conference abstracts without full-text articles, duplicate reports, and non-peer-reviewed sources were not included.

Because the available literature differs substantially across DKD, diabetes-related ED, and recurrent UTIs, the evidence was interpreted with attention to study design, population specificity, endpoint relevance, and mechanistic directness. Greater emphasis was placed on studies involving patients with diabetes, clinically defined genitourinary outcomes, prospective or interventional designs, and systematic reviews or meta-analyses. Mechanistic studies and microbiome-related evidence were used mainly to support biological plausibility and to develop the proposed conceptual framework, rather than to establish direct causality. This approach allowed us to distinguish the relatively stronger evidence base for DKD from the more limited evidence for diabetes-related ED and the mainly indirect or hypothesis-generating evidence for recurrent UTIs. Because this review was designed as a narrative mechanistic review rather than a systematic review or meta-analysis, we did not perform a formal study-selection process according to the Preferred Reporting Items for Systematic Reviews and Meta-Analyses, nor did we conduct a risk-of-bias assessment or a certainty-of-evidence assessment using the Grading of Recommendations Assessment, Development and Evaluation framework. Therefore, the evidence ratings in **Table 1** should be interpreted only as a narrative, outcome-specific appraisal of evidence directness, consistency, and disease specificity. These ratings are intended to help readers understand the relative evidence gradient across DKD, diabetes-related ED, and recurrent UTIs, and should not be interpreted as formal certainty-of-evidence grades or as implying comparable causal strength across outcomes.

**Table 1:** Summary and evidence appraisal of clinical and epidemiological evidence linking periodontitis to diabetes-related genitourinary complications.

Outcome	Study design	First author/year	Study subjects	Main finding	Main limitation	Evidence directness	Evidence strength rating
DKD	Cross-sectional study	Nair et al. (2022)	T2DM, n = 500	Chronic periodontitis was associated with a higher prevalence of microalbuminuria.	A cross-sectional design cannot establish temporal sequence or causality.	Direct for renal outcome; observational	★★★ Moderate
DKD	Cross-sectional study	Tanaka et al. (2019)	T2DM, n = 2,302	Urinary albumin excretion was significantly associated with multiple periodontal parameters.	Observational design with possible residual confounding.	Direct for renal outcome; observational	★★★ Moderate
DKD	Prospective cohort study	Mikami et al. (2025)	Poorly controlled T2DM	Periodontitis severity and periodontal inflammatory burden were significantly associated with subsequent eGFR decline.	Further validation in independent cohorts is needed.	Direct for renal outcome; prospective	★★★ Moderate
DKD	Nationwide cohort study	Kusama et al. (2025)	Middle-aged patients with T2DM; 6-year follow-up	Periodontal care was associated with a 32%–44% lower risk of dialysis initiation.	Potential care-related bias and residual confounding in database-based research.	Direct for renal outcome; observational cohort	★★★ Moderate
DKD	Meta-analysis	Yang et al. (2024)	CKD/periodontitis-related studies	Chronic periodontitis was significantly associated with increased CKD risk.	Most endpoints were not DKD-specific, and heterogeneity was substantial.	Partly direct; CKD rather than DKD-specific	★★★ Moderate
DKD	Systematic review and meta-analysis	López-Sanz et al. (2025)	Studies on CKD and chronic oral inflammatory diseases	A stable statistical association between periodontitis and CKD was reported.	Limited interpretability for DKD-specific outcomes.	Partly direct; CKD rather than DKD-specific	★★★ Moderate
DKD	Randomized controlled trial	Fang et al. (2015)	Patients with ESRD, 6 months	Non-surgical periodontal therapy may improve some renal function-related indicators.	The intervention population was not a typical DKD population, limiting generalizability.	Partly direct; interventional but not typical DKD population	★★★ Moderate
ED	Cross-sectional study	Matsumoto et al. (2014)	General male population	Chronic periodontal disease was significantly associated with ED.	Most evidence came from non-diabetic populations.	Indirect for diabetes-related ED	★★ Low
ED	Retrospective population-based study	Tsao et al. (2015)	Male population from a Taiwan database	Periodontitis was associated with a higher subsequent risk of ED.	Causality remains unclear.	Indirect for diabetes-related ED	★★ Low
ED	Systematic review and meta-analysis	Farook et al. (2021)	6 studies; >215,000 men	Periodontitis was associated with an approximately 2.5-fold higher risk of ED.	Heterogeneity was present, and diabetes-specific evidence was limited.	Indirect for diabetes-related ED	★★ Low
ED	Interventional studies	Eltas et al. (2013); El-Makaky et al. (2020)	Patients with ED and periodontitis	Limited interventional evidence suggests that non-surgical periodontal treatment may partially improve ED severity.	Interventional evidence remains limited, and diabetes-specific validation is lacking.	Partly direct for ED; not diabetes-specific	★★ Low
Recurrent UTI	Cross-sectional study	Xu et al. (2025)	Systemically healthy individuals; periodontitis n = 229, healthy controls n = 36	Severe periodontitis was associated with increased systemic inflammatory burden and immune dysregulation, providing mechanistic support for infection susceptibility.	A cross-sectional design cannot establish temporal sequence or causality.	Mechanistic support only; not UTI-specific	★ Very low/hypothesis-generating
Recurrent UTI	Population-based study	Li et al. (2021)	Periodontitis-related populations	Periodontitis was associated with a higher risk of Helicobacter pylori infection.	Not specific to recurrent UTI and provides only indirect support.	Indirect; extraoral infection outcome, not recurrent UTI-specific	★ Very low/hypothesis-generating
Recurrent UTI	Population-based study	Mihalčin et al. (2026)	Populations with dental and periodontal infection-related conditions	Infectious complications related to dental and periodontal infections were increased.	Not a diabetes-specific recurrent UTI study.	Indirect; infectious complications, not recurrent UTI-specific	★ Very low/hypothesis-generating
Recurrent UTI	Systematic review	Hasan et al. (2025)	Studies on periodontitis and systemic health	Periodontitis may affect remote organ homeostasis through systemic inflammation.	Direct clinical validation for recurrent UTI in patients with diabetes is lacking.	Indirect mechanistic support	★ Very low/hypothesis-generating
Overall appraisal	—	—	—	Evidence is most substantial for DKD, followed by ED, whereas recurrent UTI is currently supported mainly by indirect evidence and mechanistic inference.	Current evidence supports periodontitis more strongly as a potentially modifiable amplifying factor than as a confirmed independent cause across all endpoints.	Uneven across outcomes; most direct for DKD and most indirect for recurrent UTI	DKD: ★★★ Moderate; ED: ★★ Low; recurrent UTI: ★ Very low

Evidence strength rating: ★★★ Moderate = the most consistent evidence among the reviewed outcomes, although still largely observational; ★★ Low = limited or non-diabetes-specific evidence; ★ Very low/hypothesis-generating = indirect or mechanistic evidence without direct disease-specific validation. These ratings represent a narrative evidence appraisal and should not be interpreted as formal certainty-of-evidence ratings. To visually emphasize the evidence gradient, DKD is categorized as the most directly supported endpoint, ED as a preliminary but biologically plausible endpoint, and recurrent UTI as a highly preliminary, hypothesis-generating endpoint with no direct diabetes-specific longitudinal validation.

DKD, diabetic kidney disease; ED, erectile dysfunction; UTI, urinary tract infection; T2DM, type 2 diabetes mellitus; CKD, chronic kidney disease; ESRD, end-stage renal disease; eGFR, estimated glomerular filtration rate.

## 3 Epidemiological and clinical evidence

Current clinical and epidemiological evidence linking periodontitis to diabetes-related genitourinary complications is uneven across outcomes, with the strongest support observed for diabetic kidney disease, based on cross-sectional studies, cohort data, and meta-analyses. By contrast, the evidence for diabetes-related erectile dysfunction remains more limited and is derived largely from non-diabetic populations, whereas support for recurrent urinary tract infections in patients with diabetes is currently indirect and relies mainly on mechanistic inference and non-specific infectious outcomes rather than direct diabetes-specific clinical studies. Therefore, the following sections summarize the available evidence according to both outcome category and evidentiary strength, with caution to avoid interpreting associations as established causality (**Table 1**).

### 3.1 Periodontitis and diabetic kidney disease

Diabetes mellitus is one of the major causes of chronic kidney disease (CKD), and DKD represents a clinically important diabetes-specific renal phenotype. However, many epidemiological studies examining the relationship between periodontitis and kidney disease have used broader CKD endpoints rather than DKD-specific outcomes. Therefore, evidence derived from general CKD populations should not be directly equated with DKD-specific evidence, because CKD includes heterogeneous etiologies, risk-factor profiles, and disease trajectories. In the present review, studies conducted specifically in patients with diabetes or reporting diabetes-relevant renal outcomes, such as albuminuria, eGFR decline, or dialysis initiation, are considered the most directly relevant evidence for DKD. By contrast, broader CKD studies and meta-analyses are used as supportive renal background for biological plausibility and shared inflammatory or microvascular pathways, rather than as definitive DKD-specific evidence. At the epidemiological and clinical observation level, existing cross-sectional studies suggest that there may be an association between periodontitis and early indicators of kidney injury in patients with type 2 diabetes. A cross-sectional study conducted by Nair et al. included 500 patients with type 2 diabetes. The results showed that microalbuminuria was more common in patients with chronic periodontitis, suggesting that periodontitis may be associated with an increased risk of early kidney injury in patients with diabetes (Nair et al., 2022). Another cross-sectional study involving 2,302 patients with type 2 diabetes further found that urinary albumin excretion levels were significantly associated with multiple periodontal parameters, and this association persisted after adjusting for confounding factors, suggesting that periodontitis may be associated with early clinical markers of diabetic kidney injury (Tanaka et al., 2019).

Cohort studies have provided additional support for an association between periodontitis and adverse kidney outcomes in patients with diabetes. A prospective cohort study conducted by Mikami et al. showed that in type 2 diabetes patients with poor glycemic control, the severity of periodontitis and periodontal inflammatory burden were significantly associated with subsequent decline in estimated glomerular filtration rate (eGFR), suggesting that periodontitis may be associated with renal function deterioration in patients with diabetes (Mikami et al., 2025). Another 6-year follow-up cohort study based on a national medical database further found that patients with type 2 diabetes who received periodontal care had a dialysis initiation risk of about 32%–44% lower than those who did not receive such care, suggesting that standardized periodontal management may be associated with improved renal outcomes in patients with diabetes (Kusama et al., 2025).

Broader CKD-focused systematic reviews and meta-analyses provide additional supportive context for kidney-related biological plausibility, although they should not be interpreted as DKD-specific confirmation. A meta-analysis including 22 studies showed a significant association between chronic periodontitis and the risk of CKD (Yang F. et al., 2024). Another systematic review indicated a stable statistical association between periodontitis and CKD, suggesting that the two diseases may share or interact through mechanisms such as systemic inflammation, immune dysregulation, and oxidative stress (López-Sanz et al., 2025). In addition, an intervention study in patients with end-stage renal disease suggested that non-surgical periodontal treatment may improve periodontal status, circulating inflammatory markers, and selected renal-related biochemical indicators (Fang et al., 2015). Overall, existing epidemiological and clinical studies suggest that periodontitis may be associated with DKD progression and may serve as a potential amplifying factor in susceptible patients with diabetes. However, most available studies remain observational, and these findings should be interpreted as supporting association and biological plausibility rather than proving a direct causal relationship.

### 3.2 Periodontitis and diabetes-related erectile dysfunction

Current research suggests a relatively consistent epidemiological association between periodontitis and ED. However, direct evidence in patients with diabetes remains relatively limited. Existing studies are mostly from the general male population, and their results provide more indirect support for understanding the inflammatory and endothelial pathways potentially relevant to diabetes-related ED. Cross-sectional studies have shown a significant association between chronic periodontitis and ED, suggesting that poor periodontal health is associated with a higher likelihood of ED (Matsumoto et al., 2014). A retrospective population study based on a Taiwanese population database further showed that patients with periodontitis had a higher subsequent risk of ED (Tsao et al., 2015). A systematic review and meta-analysis included more than 215,000 men in six studies, and the results showed that patients with periodontitis had an approximately 2.5-fold increased risk of developing ED (Farook et al., 2021). A small number of existing studies suggest that systemic inflammation and vascular endothelial dysfunction may provide a biologically plausible link between periodontitis and ED. For example, changes such as elevated levels of inflammatory mediators, such as interleukin-6 (IL-6) and tumor necrosis factor-α (TNF-α), and decreased nitric oxide (NO) bioavailability may be relevant to ED pathophysiology by impairing endothelium-dependent relaxation and penile cavernosal perfusion (Li et al., 2022; Giagulli et al., 2024). Limited interventional evidence, including a randomized controlled trial and a small clinical study, suggests that non-surgical periodontal treatment may be associated with improvements in ED-related scores among patients with periodontitis and ED (Eltas et al., 2013; El-Makaky et al., 2020). However, these findings should be interpreted cautiously because the available interventional evidence remains limited, and diabetes-specific interventional data are still insufficient. Overall, compared with DKD, the relationship between periodontitis and diabetes-related ED should be interpreted more cautiously. Current evidence supports a possible association and biological plausibility, but does not establish that periodontitis independently causes ED or that periodontal treatment improves diabetes-related ED. Diabetes-specific prospective and interventional studies are still needed to determine whether periodontitis is independently associated with ED risk or severity in patients with type 2 diabetes.

### 3.3 Periodontitis and recurrent urinary tract infections in patients with diabetes

Patients with diabetes have a significantly increased risk of UTIs due to the hyperglycemic environment, impaired immune function, and altered urinary tract defense mechanisms, and are more prone to recurrent infections (Geerlings, 2016). Recent studies suggest that periodontitis, as a chronic systemic inflammatory disease, may be linked to infection susceptibility in patients with diabetes by increasing systemic inflammatory burden and disrupting host immune homeostasis (Pari et al., 2023; Zhao et al., 2023). Cross-sectional studies have shown that patients with severe periodontitis have higher systemic inflammatory burden and immune dysregulation; at the same time, original studies suggest that their peripheral phagocytic cell function is impaired, and these changes may weaken the host’s defense against bacterial infection (Naiff et al., 2021; Xu et al., 2025). Population studies have also suggested that periodontitis and related dental/periodontal foci are associated with some extraoral infectious outcomes and serious infectious complications. Currently, few prospective studies have directly assessed recurrent UTIs in patients with diabetes as an outcome in relation to periodontal status. Population-based studies have suggested that periodontitis and dental/periodontal infectious foci may be associated with selected extraoral infectious outcomes, including Helicobacter pylori infection and serious infectious complications (Li et al., 2021; Mihalčin et al., 2026). However, these findings are not specific to recurrent UTIs in patients with diabetes and should be interpreted only as indirect support. The systematic review further points out that periodontitis-related chronic inflammation can affect the homeostasis of distal organs through systemic inflammation and immune imbalance (Hasan et al., 2025). Potential mechanisms may include oral bacteremia and the microbial transfer hypothesis, that is, after periodontal pathogens and their related inflammatory mediators enter the bloodstream, they can induce immune activation in distal mucosal tissues and affect barrier function (Yamazaki and Kamada, 2024). In addition, the changes in TNF-α, IL-6, and high-sensitivity C-reactive protein after periodontal treatment further support that periodontal inflammation can affect the systemic inflammatory load, suggesting that periodontal inflammation may be biologically relevant to infection susceptibility rather than providing direct evidence for recurrent UTI occurrence in patients with diabetes (Cardisciani et al., 2025). Although clinical studies directly examining the relationship between periodontitis and recurrent UTIs in patients with diabetes remain scarce, existing epidemiological and mechanistic studies suggest that periodontitis-related systemic inflammation, immune dysregulation, and microbial dissemination may provide a hypothesis-generating basis for this association. Therefore, among the three outcomes discussed in this review, recurrent UTIs currently represent the most indirect and hypothesis-generating endpoint, and direct longitudinal studies in patients with diabetes and well-characterized periodontal status are still needed.

## 4 Core mechanism pathways

**Figure 1:**
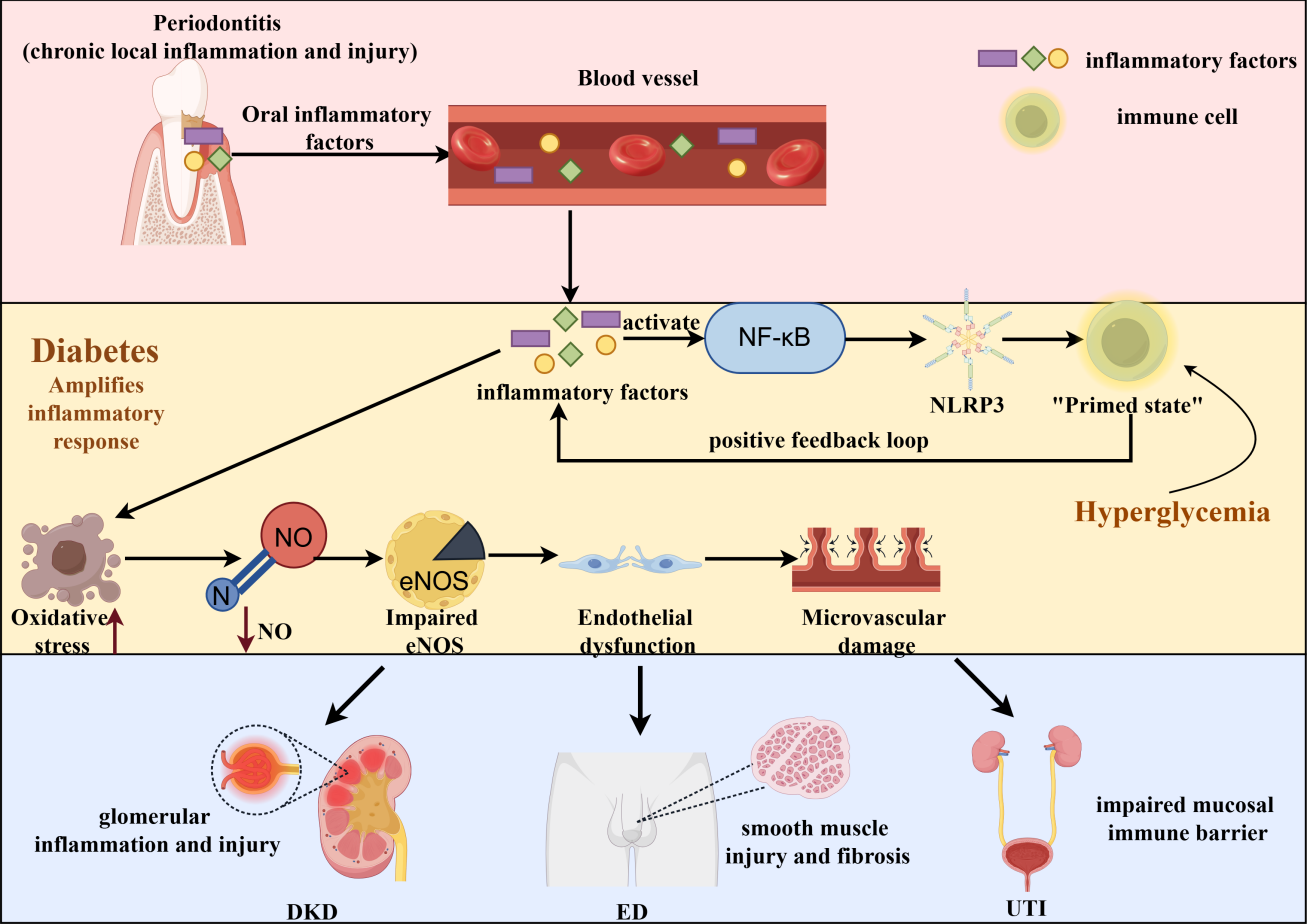
Schematic illustration of the potential mechanisms connecting periodontitis with diabetes-related genitourinary complications. In diabetes, periodontitis-related systemic inflammation is synergistically enhanced by hyperglycemia-associated immune activation, and together with endothelial dysfunction and microvascular damage, may contribute to biological pathways associated with diabetic kidney disease (DKD), diabetes-related erectile dysfunction (ED), and susceptibility to urinary tract infections (UTIs). (The arrows pointing to DKD, ED, and UTI indicate integrated biological effects or plausible mechanistic links of the above mechanistic pathways, rather than the action of any single mediator alone. These arrows should not be interpreted as indicating proven causality or equivalent evidence strength across outcomes; current clinical evidence is strongest for DKD, more preliminary for diabetes-related ED, and most hypothesis-generating for recurrent UTIs.).

**Figure 2:**
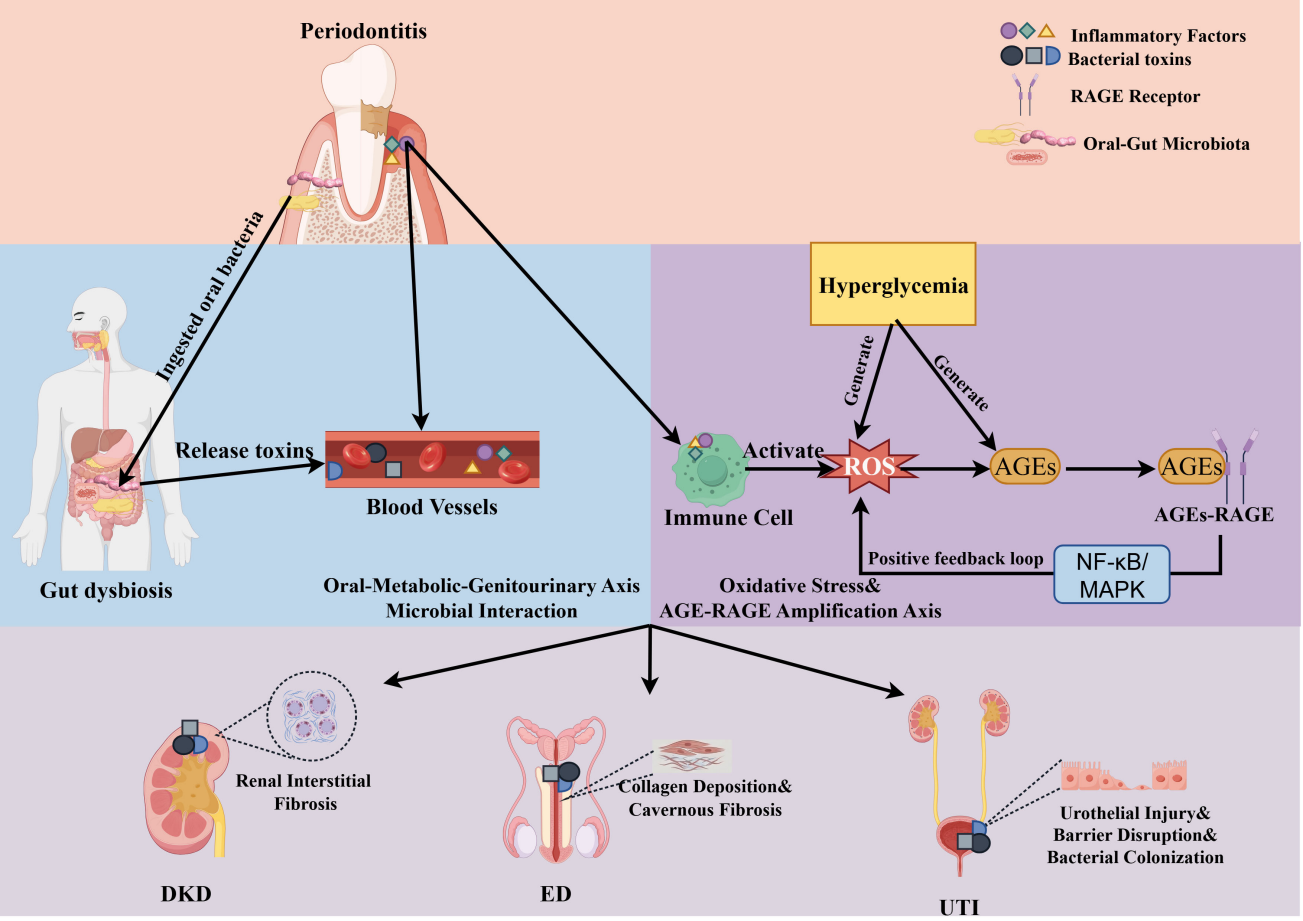
Proposed amplifying mechanisms linking periodontitis to diabetes-related genitourinary complications. Periodontitis-associated oral dysbiosis may promote bacterial translocation, gut dysbiosis, and systemic dissemination of microbial products, thereby enhancing systemic inflammation. In the diabetic state, hyperglycemia-induced oxidative stress and activation of the AGE–RAGE signaling axis further amplify inflammatory responses, endothelial injury, and tissue fibrosis. Through this oral–metabolic–genitourinary axis, these interacting mechanisms may provide biological plausibility for links with diabetic kidney disease (DKD), diabetes-related erectile dysfunction (ED), and recurrent urinary tract infections (UTIs), with the strongest support for renal outcomes and more indirect support for ED and recurrent UTIs. (The arrows pointing to DKD, ED, and UTI indicate integrated biological effects or plausible mechanistic links of the above mechanistic pathways, rather than the action of any single mediator alone. This model is intended to illustrate biological plausibility and hypothesis generation rather than a proven causal sequence. In particular, direct evidence for oral–gut–genitourinary microbial transmission or for a causal effect of periodontitis on ED or recurrent UTIs in patients with diabetes remains limited.).

Several interconnected pathways may provide biological plausibility for the observed associations between periodontitis and diabetes-related genitourinary complications. These pathways should be interpreted as mechanistic hypotheses rather than evidence of direct causality. As summarized in **Figure 1**, periodontitis-related systemic inflammation may converge with diabetes-associated immune activation, endothelial dysfunction, and microvascular injury, forming a proposed inflammation–microvascular injury–organ vulnerability pathway. This pathway is most directly supported in relation to renal outcomes, whereas its extension to diabetes-related ED and recurrent UTIs remains more inferential. Oxidative stress, AGE–RAGE signaling, and microbiome interactions may further amplify this proposed framework, as discussed below and illustrated in **Figure 2**.

### 4.1 Chronic systemic inflammation as a potential amplifying pathway

Chronic systemic inflammation represents a biologically plausible pathway linking periodontitis with diabetes-related metabolic and vascular injury. In this section, we discuss how periodontitis-related inflammatory mediators may overlap with hyperglycemia-associated immune activation. This pathway is most directly relevant to renal inflammatory and microvascular injury, whereas its relevance to diabetes-related ED and recurrent UTIs remains more inferential. Periodontitis is a chronic inflammatory disease driven by microbial imbalance. During the continuous inflammatory response in periodontal tissues, a large number of inflammatory mediators can enter the systemic circulation, thus forming a persistent low-grade systemic inflammatory state. Studies have shown that the levels of multiple inflammatory factors in the plasma of patients with periodontitis are significantly elevated, including interleukin-6, tumor necrosis factor-α, and C-reactive protein. These inflammatory factors not only participate in local inflammatory responses but can also further amplify systemic inflammatory responses by activating the nuclear factor κB (NF-κB) signaling pathway and the NOD-like receptor protein 3 (NLRP3) inflammasome (Preshaw et al., 2012; Zhao et al., 2022).

In the context of diabetes, this inflammatory response can be further amplified. A hyperglycemic environment can induce immune cells to be in a “pre-activated state,” enhancing the responsiveness of monocytes and macrophages to inflammatory stimuli, thereby forming a persistent chronic inflammatory microenvironment (Lisco et al., 2022; Lee et al., 2024). Studies have shown that a hyperglycemic state can promote the activation of the NF-κB signaling pathway and enhance the expression of the NLRP3 inflammasome, thereby increasing the release of inflammatory mediators and contributing to inflammatory tissue injury (Hotamisligil, 2017). Therefore, the periodontitis-associated inflammatory response in patients with diabetes is superimposed on hyperglycemia-related inflammation, forming a synergistic amplification effect of inflammation (Preshaw et al., 2012).

This persistent inflammatory state may be relevant to several diabetes-related target-organ processes. In the kidneys, inflammatory mediators may contribute to mesangial cell activation, glomerular inflammation, and renal microvascular injury relevant to DKD (Navarro-González and Mora-Fernández, 2008). In the penile corpora cavernosa, inflammatory factors may impair endothelial-dependent vasodilation and smooth muscle homeostasis, providing a biologically plausible link with ED (Maiorino et al., 2014). In the urinary tract, chronic inflammation may affect mucosal immune defense and host antibacterial responses, thereby providing indirect mechanistic support for susceptibility to recurrent UTIs in patients with diabetes (Geerlings, 2016). Therefore, periodontitis-related systemic inflammation may provide a plausible inflammatory amplification pathway in the diabetic milieu. This pathway is most directly supported in relation to renal inflammatory and microvascular injury, whereas its relevance to diabetes-related ED and recurrent UTIs remains more indirect and requires further disease-specific validation.

### 4.2 Microvascular and endothelial dysfunction

Besides chronic systemic inflammation, microvascular and endothelial dysfunction may represent a biologically plausible pathway linking periodontitis with diabetes-related genitourinary complications. Endothelial dysfunction is the molecular basis of microvascular injury, while microvascular lesions are its important manifestation at the organ level. The vascular endothelium plays a core role in maintaining vascular homeostasis and tissue perfusion, among which NO is one of the most important endothelial-derived vasodilators. Studies have shown that periodontitis-related inflammation and oxidative stress can interfere with NO metabolism and reduce its bioavailability, thereby contributing to impaired endothelial-dependent vasodilatory function (Lima et al., 2024). In addition, periodontal pathogenic bacterial infection can also disrupt vascular endothelial homeostasis by affecting the activity of endothelial nitric oxide synthase (eNOS) and NO signaling pathways, potentially contributing to endothelial dysfunction and increased vascular permeability (Dhungana et al., 2025). This vascular-inflammatory interpretation is also supported by evidence from other vascular disease contexts. A recent mini-review on periodontitis and peripheral artery disease summarized a modest association between periodontal disease and peripheral artery disease and highlighted systemic inflammation, endothelial activation, circulating inflammatory mediators, oral pathogens, and pathogen-specific immune responses as potential connecting mechanisms, particularly in individuals with shared cardiometabolic risk factors such as diabetes, smoking, hypertension, older age, and CKD (De Falco et al., 2026). Although this evidence does not directly address diabetes-related genitourinary outcomes, it supports the broader concept that periodontal inflammation may contribute to systemic endothelial and microvascular vulnerability under metabolically high-risk conditions.

In the context of diabetes, microvascular damage is more pronounced. Long-term hyperglycemia can damage vascular endothelial cells and cause changes in microvascular structure through oxidative stress and inflammatory responses. In the kidneys, diabetes-related endothelial dysfunction and microvascular injury may contribute to abnormal glomerular structure, impaired local perfusion, and inflammatory injury relevant to DKD (Forbes and Cooper, 2013; Młynarska et al., 2024; Wang and He, 2024). Similar microvascular lesions can also occur in the penile corpora cavernosa. Erectile function depends on intact vascular endothelial function and smooth muscle relaxation response mediated by the NO–cyclic guanosine monophosphate (cGMP) signaling pathway. Diabetes and chronic inflammation can both lead to damage to the NO signaling pathway and reduce blood flow perfusion in the corpora cavernosa, thereby inducing changes in the structure of corpora cavernosa smooth muscle cells and fibrotic remodeling, potentially contributing to the vascular component of ED (Defeudis et al., 2022). In addition, microvascular ischemia and nerve damage also play an important role in lower urinary tract dysfunction in diabetes. Studies have shown that diabetes-related microvascular lesions can lead to reduced bladder blood flow and affect the function of bladder wall nerves and smooth muscle, thereby inducing detrusor muscle remodeling and decreased bladder sensory function, forming diabetic cystopathy (Hughes et al., 2022; He et al., 2024). Local bladder ischemia and neuro-microvascular injury may contribute to bladder emptying dysfunction and urinary retention, which could weaken urinary tract defense and create conditions favorable for bacterial colonization, thereby potentially increasing susceptibility to recurrent UTIs in patients with diabetes (Papp and Zimmern, 2023). Therefore, endothelial dysfunction and microvascular injury may provide a shared biological pathway through which periodontal inflammation and diabetes-related metabolic abnormalities could converge. This pathway is most directly supported in relation to DKD and is biologically plausible for diabetes-related ED, whereas its relevance to recurrent UTIs remains more indirect and may be mediated through bladder dysfunction, impaired urinary defense, or neuro-microvascular injury.

### 4.3 Oxidative stress and the AGE–RAGE axis

As illustrated in **Figure 2**, oxidative stress, AGE–RAGE signaling, and microbiome-related inflammatory signals may form a proposed amplification loop within the oral–metabolic–genitourinary axis. In this model, reactive oxygen species (ROS) generation may enhance AGE–RAGE-related inflammatory signaling, while microbiome-derived stimuli may further reinforce systemic inflammation and metabolic vascular stress. This loop should be interpreted as a biologically plausible framework rather than a proven causal sequence.

Oxidative stress may represent one of the biologically plausible mechanisms linking periodontitis with diabetes-related microvascular injury. It is important to emphasize that oxidative stress and AGE–RAGE signaling are closely interconnected with inflammation and microvascular dysfunction. They may reinforce systemic inflammatory responses, endothelial dysfunction, and fibrotic remodeling within the proposed oral–metabolic–genitourinary axis. In the diabetic state, long-term hyperglycemia can increase mitochondrial electron transport chain activity and promote excessive ROS production, thereby contributing to mitochondrial dysfunction, lipid peroxidation, and DNA damage (Zhang Z. et al., 2023). In addition, periodontitis-related inflammatory responses may further increase ROS production through immune-cell activation and oxidative-stress pathways, thereby contributing to a persistent oxidative-stress state (Shang et al., 2023). Under high oxidative stress, glucose and its metabolites can form AGEs through non-enzymatic glycation reactions. AGEs may accumulate in diabetic tissues and, through binding to RAGE, activate inflammation- and fibrosis-related signaling pathways, including nuclear factor κB (NF-κB) and mitogen-activated protein kinase (MAPK) pathways. These processes may further reinforce inflammatory responses and tissue injury (Chen et al., 2024). Therefore, the AGE–RAGE signaling axis may amplify oxidative-stress responses and contribute to extracellular matrix deposition and structural remodeling.

In diabetes-related genitourinary target organs, these processes may be relevant to structural remodeling and microvascular injury. In the kidneys, AGE–RAGE signaling may contribute to glomerular mesangial cell activation, renal interstitial fibrosis, and microvascular injury relevant to DKD (Forbes and Cooper, 2013). In the penile corpus cavernosum tissue, oxidative stress and AGEs accumulation may contribute to smooth muscle dysfunction, collagen deposition, and cavernosal fibrosis, providing a biologically plausible link with ED-related vascular impairment (Rhee and Kim, 2018; Defeudis et al., 2022). Meanwhile, thickening of the microvascular basement membrane and changes in vascular wall structure are also important features of diabetic microvascular complications, which further aggravate local ischemia and tissue damage (Forbes and Cooper, 2013). In addition, oxidative stress and the AGE–RAGE signaling axis may also affect the function of the urothelial barrier. Studies have shown that the binding of AGE and RAGE can activate inflammatory signaling pathways such as NF-κB and promote the generation of ROS, thereby inducing inflammatory responses and tissue damage (Rungratanawanich et al., 2021). In the diabetic context, high-glucose-associated oxidative and inflammatory signaling, potentially including AGE–RAGE-related pathways, may participate in urothelial damage and barrier dysfunction (Rungratanawanich et al., 2021; Song et al., 2022). Experimental and clinical studies further suggest that diabetes may impair bladder urothelial defense mechanisms and affect tight junction-related proteins, thereby weakening urinary tract mucosal barrier integrity and local immune defense (Odom et al., 2022; Song et al., 2022; Mohanty et al., 2024; Schwartz et al., 2024). Such impaired barrier dysfunction could theoretically facilitate bacterial adhesion and colonization, thereby providing indirect mechanistic support for increased UTI susceptibility in patients with diabetes. Therefore, oxidative stress and the AGE–RAGE signaling axis may play a key amplifying role in the relationship between periodontitis-related inflammation and diabetes-related genitourinary complications. Overall, oxidative stress and AGE–RAGE signaling provide a biologically plausible amplification loop linking periodontal inflammation with diabetes-related inflammatory and microvascular injury. This pathway is most directly relevant to DKD and is biologically plausible for diabetes-related ED, whereas its connection with recurrent UTIs remains indirect and hypothesis-generating.

### 4.4 Research progress on the mechanisms of microbiome interactions in the oral–metabolic–genitourinary axis

In addition to systemic inflammation and microvascular injury, microbiome interactions may provide another biologically plausible but still largely hypothesis-generating link between periodontitis and diabetes-related genitourinary complications. However, direct longitudinal evidence demonstrating oral–gut–genitourinary microbial transmission or causal effects on diabetes-related genitourinary outcomes remains limited.

Periodontitis is a chronic inflammatory disease driven by an imbalance in the oral microbiota. Numerous periodontal pathogens can colonize periodontal pockets and enter the bloodstream when the gingival epithelial barrier is compromised (de Jongh et al., 2023; Zhang et al., 2024). Studies have shown that periodontal infection can lead to transient bacteremia, allowing oral bacteria and microbial products to enter the systemic circulation and potentially interact with distant tissues (Hajishengallis and Chavakis, 2021). Furthermore, multiple studies have detected periodontal-associated bacterial DNA in cardiovascular tissues such as atherosclerotic plaques, and recent reviews have summarized evidence of oral pathogens in other distal organ-related diseases, suggesting that hematogenous dissemination of oral microbial components may be biologically relevant to systemic diseases (Rao et al., 2021; Xi et al., 2024). Therefore, oral bacteremia is considered one of the important mechanisms by which periodontitis affects systemic health.

In recent years, an increasing number of studies have proposed the concept of the “oral–gut axis.” Patients with periodontitis continuously swallow large amounts of saliva containing oral bacteria daily. Some oral microorganisms may reach the gastrointestinal tract and, under susceptible conditions, may influence gut microbial composition and mucosal immune responses (Bao et al., 2022; Yamazaki, 2023). Related studies have shown that imbalances in the oral microbiota can not only affect local periodontal tissues but may also promote the development of various systemic diseases, including metabolic and inflammatory diseases, through gut microbiota dysbiosis (Xi et al., 2024). Therefore, oral–gut microbial interactions may represent a potential bridge between oral inflammation and systemic metabolic or inflammatory disorders.

Gut microbiota dysbiosis is considered to play an important role in the development and progression of various metabolic diseases and chronic kidney disease. Existing studies have shown that an imbalanced gut microbiota can increase the production of uremic toxins and inflammation-related metabolites such as indoxyl sulfate, p-cresyl sulfate, and trimethylamine N-oxide (Alobaidi, 2025; Tsuji et al., 2024). After entering the bloodstream, these substances may contribute to kidney injury and chronic kidney disease progression by inducing chronic inflammation, oxidative stress, endothelial dysfunction, and fibrosis (Alobaidi, 2025; Tsuji et al., 2024). In addition, longitudinal population studies have also suggested that dynamic changes in gut microbiota composition are associated with the persistence or progression of chronic kidney disease, providing further population evidence for the role of the gut–kidney axis in kidney injury (Atzeni et al., 2024). In terms of the kidneys, studies have found that oral microbiota imbalance may be linked to kidney disease partly through gut microbiota-mediated inflammatory and metabolic pathways (Bao et al., 2022; Li et al., 2025). Besides kidney damage, gut microbiota and its metabolic abnormalities may also be involved in the development of ED in diabetes (Zhang Y. et al., 2023; Su et al., 2024). The maintenance of erectile function depends on intact vascular endothelial function, normal nitric oxide bioavailability, and corpus cavernosum homeostasis, while diabetes is often accompanied by microvascular damage, chronic low-grade inflammation, and abnormal tissue remodeling (Ma et al., 2024; Hostnik et al., 2025). Therefore, gut microbiota-related metabolic disturbances may be biologically relevant to ED by influencing endothelial function, nitric oxide bioavailability, and local tissue remodeling (Ma et al., 2024; Su et al., 2024). Furthermore, gut microbiota is considered an important source of urinary tract microbiota; alterations in gut microbiota structure may influence the reservoir of potential uropathogens and may be relevant to urinary tract colonization and UTI susceptibility (Choi et al., 2024; Iqbal et al., 2024). Therefore, although direct evidence is currently limited, the microbial interactions within the oral–gut–genitourinary system may constitute a potential biological basis for the “oral–metabolic–genitourinary axis” and provide a new research perspective for understanding the distal links between periodontitis and diabetes-related genitourinary complications.

Nevertheless, the microbiome component of the proposed oral–metabolic–genitourinary axis should be interpreted with caution. Most available studies are cross-sectional, preclinical or experimental, or only indirectly related to diabetes-related genitourinary outcomes, and few have simultaneously profiled oral, gut, and urinary microbiota in longitudinal cohorts of patients with diabetes (Worby et al., 2022; Kunath et al., 2024). Microbiome findings are also highly susceptible to confounding by diet, antibiotic exposure, glycemic control, renal function, sex, age, medication use, and sampling or sequencing methods (Falony et al., 2016; Karstens et al., 2018). In addition, detection of microbial DNA or taxonomic overlap does not necessarily demonstrate viable bacterial translocation, functional activity, or causal involvement in organ injury (Emerson et al., 2017; Eisenhofer et al., 2019). Therefore, current microbiome evidence should be viewed primarily as hypothesis-generating support for biological plausibility rather than as proof of a causal oral–gut–genitourinary mechanism.

## 5 Modifiers of the proposed oral–metabolic–genitourinary axis

In addition to the core mechanisms discussed above, including inflammation, microvascular injury, oxidative stress, AGE–RAGE signaling, and microbiome interactions, host genetic susceptibility, metabolic status, lifestyle factors, and microecological background may further modify the proposed relationship between periodontitis and diabetes-related genitourinary outcomes.

Genetic and host susceptibility factors should also be considered as upstream modifiers of this proposed axis. The host response to periodontal dysbiosis is not determined only by microbial burden, but also by genetically regulated innate immune recognition, inflammatory signaling, and tissue-destructive responses (Brodzikowska and Górski, 2022). Pattern-recognition receptors, particularly Toll-like receptors, play a central role in sensing periodontal microbial components and initiating downstream inflammatory pathways (Jin et al., 2016; Brodzikowska and Górski, 2022). Among these receptors, Toll-like receptor 4 (TLR4) is especially relevant because it links bacterial lipopolysaccharide recognition with NF-κB-mediated inflammatory activation (Jin et al., 2016). Previous studies and meta-analyses have examined associations between TLR4 polymorphisms and periodontitis susceptibility, although findings may vary across populations and polymorphic loci (Jin et al., 2016; Cavalla et al., 2025). More recently, an exploratory study of patients with end-stage renal disease suggested that the TLR4 rs2149356 polymorphism may be associated with periodontal and renal parameters, supporting a possible link among genetic predisposition, host–microbe interactions, periodontal inflammation, and renal vulnerability (Abou-Bakr et al., 2025). However, this evidence should be interpreted cautiously, because current genetic studies remain limited and do not establish causal modification of diabetes-related genitourinary outcomes. Future studies integrating host genomics, oral–gut–genitourinary microbiome profiling, inflammatory biomarkers, and clinical outcomes are needed to clarify whether genetic susceptibility modifies the proposed oral–metabolic–genitourinary axis (Lafleur et al., 2023).

Obesity and metabolic syndrome can be considered important synergistic factors between periodontitis and diabetes-related genitourinary complications. In an obese state, adipose tissue can continuously release pro-inflammatory factors and aggravate insulin resistance, thereby amplifying the periodontitis-related systemic inflammatory response; while there is also a mutually reinforcing relationship between the components of metabolic syndrome and periodontitis. This overlap of inflammation and metabolic abnormalities may increase susceptibility to kidney, vascular, and other genitourinary complications (Barnawi et al., 2024; Reytor-González et al., 2024). On the other hand, glycemic control level is also a key factor affecting the strength of this association. Existing evidence shows that elevated glycated hemoglobin A1c (HbA1c) is associated with worse periodontal condition, and periodontal treatment can improve HbA1c levels to some extent, suggesting that hyperglycemia may aggravate periodontal inflammation and may strengthen the inflammatory and microvascular background associated with diabetes-related genitourinary complications (Simpson et al., 2022; Umezaki et al., 2025).

Smoking and related unhealthy lifestyle habits may also enhance the systemic adverse effects of periodontitis. Smoking not only impairs blood supply to periodontal tissues and weakens the host immune response but also exacerbates diabetes-related vascular damage by promoting oxidative stress and endothelial dysfunction, thereby potentially increasing systemic vascular and inflammatory vulnerability relevant to diabetes-related genitourinary outcomes. Smoking is considered an important risk factor for periodontitis, and smoking cessation and lifestyle interventions have potential significance for improving oral and systemic health (Preshaw et al., 2012; Leite et al., 2018). Vitamin D deficiency is also a concern. Recent studies suggest that vitamin D deficiency is associated with increased severity of periodontitis and may further affect immune regulation, bone metabolism, and inflammatory status in the context of diabetes and chronic kidney disease, thereby potentially modifying the association between periodontitis and diabetes-related complications (MaChado et al., 2020; Park et al., 2024). Finally, gut microbiota dysbiosis may constitute another microecological modifier. As discussed above, oral microbiota imbalance may influence gut microbial composition and host immune-metabolic homeostasis through the oral–gut axis, while gut microbiota dysbiosis may be associated with systemic inflammation, metabolic dysregulation, and immune imbalance (Xi et al., 2024; Yamazaki and Kamada, 2024). However, in the context of diabetes-related genitourinary complications, this modifying role remains mainly hypothesis-generating and should not be interpreted as direct evidence of a causal oral–gut–genitourinary pathway. In summary, these modifying and synergistic factors should not be regarded as independent pathogenic mechanisms, but rather as contextual factors that may strengthen the proposed association between periodontitis and diabetes-related genitourinary complications by influencing inflammation, oxidative stress, metabolic imbalance, immune regulation, and host susceptibility.

## 6 Clinical translational significance and management implications

From a clinical perspective, periodontitis should be viewed as a potentially modifiable inflammatory condition that may be relevant to the broader management of patients with diabetes, rather than as a confirmed independent risk factor or therapeutic target for diabetes-related genitourinary complications. First, the improvement of glycemic control through periodontal treatment has been supported by relatively stable evidence. Recent systematic reviews and re-evaluations show that in patients with both diabetes and periodontitis, standardized non-surgical periodontal treatment may lead to a significant and clinically meaningful decrease in HbA1c, suggesting that periodontal treatment may improve metabolic control partly by reducing systemic inflammatory burden (Simpson et al., 2022; López-Valverde and Rueda, 2024). However, these metabolic benefits should not be interpreted as direct evidence that periodontal treatment prevents or improves diabetes-related genitourinary complications.

Second, existing research suggests that periodontal treatment may also have potential benefits for renal outcomes. A systematic review and meta-analysis showed that periodontal treatment may improve the glomerular filtration rate (GFR) in patients with chronic kidney disease, suggesting that controlling periodontitis may help improve some renal function-related indicators (da Silva et al., 2021). In addition, several review studies in recent years have suggested that there is a close bidirectional relationship between chronic kidney disease and periodontitis. Periodontitis-related systemic inflammation, oxidative stress, and oral microbial dysbiosis may be associated with renal function impairment and disease progression. Therefore, active control of periodontal inflammatory burden may help improve systemic inflammatory status and may have potential relevance to renal risk management, but whether it can directly modify DKD progression remains to be confirmed (Baciu et al., 2023; Chapple et al., 2025).

For ED, current clinical evidence remains relatively limited and is not primarily diabetes-specific. Limited interventional evidence, including a randomized controlled trial, suggests that erectile function scores may improve after periodontal treatment in patients with periodontitis and ED (Eltas et al., 2013). At the same time, systematic reviews and meta-analyses also show a stable epidemiological association between periodontitis and ED. Therefore, whether periodontal treatment has an adjunctive role in diabetes-related ED risk management remains uncertain and requires further verification in diabetic populations (Farook et al., 2021). In addition, probiotic and microecological regulation therapy has gradually attracted attention. Recent systematic reviews and meta-analyses on patients with type 2 diabetes and periodontitis suggest that adjunctive probiotic treatment may improve IL-6 levels and some periodontal clinical parameters, suggesting that microbial regulation may represent a potential adjunctive approach for reducing periodontal inflammatory burden, rather than a proven strategy for modifying diabetes-related genitourinary outcomes (Yuqi et al., 2025). For recurrent UTIs in patients with diabetes, the clinical implications of periodontal management remain even more exploratory, because current evidence is largely indirect and does not establish that periodontal treatment can reduce UTI recurrence.

Based on the above evidence, clinical management may benefit from greater multidisciplinary collaboration. Collaboration among dentistry, endocrinology, nephrology, and urology may help identify periodontitis in patients with diabetes at an early stage and incorporate oral health management into the broader chronic disease management framework. Existing research has emphasized that the bidirectional relationship between diabetes and periodontitis suggests that oral health should not be regarded as an isolated issue, but may be considered a supportive component of comprehensive diabetes management (Preshaw et al., 2012). Nevertheless, periodontal care should be viewed as complementary to established strategies for glycemic control, renal protection, cardiovascular risk reduction, infection prevention, and urological management, rather than as a substitute for these approaches.

## 7 Limitations

Several limitations should be acknowledged. First, this article is a narrative mechanistic review rather than a systematic review or meta-analysis. Although the literature search was structured, we did not perform formal PRISMA-based study selection, risk-of-bias assessment, meta-analysis, or GRADE-based certainty assessment. Therefore, the evidence ratings in **Table 1** should be interpreted as a narrative appraisal of evidence directness, consistency, and disease specificity rather than as formal evidence grades.

Second, the evidence base is uneven across outcomes. Kidney-related evidence is currently the most direct, particularly for albuminuria, eGFR decline, and dialysis-related outcomes in patients with diabetes. However, some renal evidence is derived from broader CKD populations and should be viewed as supportive rather than DKD-specific confirmation. Evidence for diabetes-related ED remains preliminary and is largely extrapolated from non-diabetic or mixed populations, whereas recurrent UTI is supported mainly by indirect mechanistic evidence and should be considered highly hypothesis-generating.

Third, residual confounding remains likely. Glycemic control, obesity, smoking, renal function, antibiotic exposure, medication use, oral hygiene behaviors, socioeconomic status, diet, and access to dental or medical care may influence both periodontal status and diabetes-related genitourinary outcomes. Future prospective cohorts, randomized trials, and multi-omics studies using standardized periodontal definitions and clinically meaningful genitourinary endpoints are needed.

## 8 Conclusions and future research directions

Current evidence suggests that periodontitis may act as a potential amplifier of selected diabetes-related genitourinary complications through overlapping pathways involving systemic inflammation, endothelial dysfunction, and microvascular injury. Among the outcomes discussed, DKD currently represents the most evidence-supported condition, particularly when diabetes-specific renal endpoints such as albuminuria, eGFR decline, and dialysis initiation are considered. However, findings from broader CKD populations should be interpreted as supportive renal background rather than definitive DKD-specific confirmation. Evidence for diabetes-related ED remains preliminary and is derived largely from non-diabetic or mixed populations, whereas evidence for recurrent UTIs in patients with diabetes remains mainly mechanistic, indirect, and highly hypothesis-generating. Therefore, periodontitis is currently more appropriately considered a potentially modifiable amplifying factor for diabetes-related genitourinary complications rather than an independent confirmed causal factor for these endpoints. Based on this evidence, the “oral–metabolic–genitourinary axis” conceptual framework proposed in this review may help to understand the potential links between periodontitis, diabetes, and diabetes-related genitourinary outcomes at a holistic level. However, this framework should be interpreted as a conceptual model rather than proof of a causal pathway. It may provide a basis for future investigation of periodontal status as a potentially modifiable component of multidisciplinary diabetes care, but it does not establish periodontal treatment as a proven strategy for preventing or improving DKD, diabetes-related ED, or recurrent UTIs.

Although existing research has suggested potential associations among periodontitis, systemic inflammation, metabolic dysfunction, and diabetes-related genitourinary complications from epidemiological, mechanistic, and limited interventional perspectives, several important research gaps remain in this field. First, large-scale prospective cohort studies are still needed to further clarify the temporal sequence, dose-response relationship, and the influence of potential confounding factors between periodontitis and diabetes-related genitourinary complications. Second, current evidence regarding the benefits of periodontal treatment is mainly based on small- to medium-sized sample studies and surrogate outcomes. Future research requires higher-quality randomized controlled trials to determine whether periodontal treatment can meaningfully influence clinical endpoints such as glycemic control, renal function outcomes, erectile function, and UTI recurrence. Meanwhile, with the deepening of research on the oral–gut axis and systemic inflammation, microbiome studies may help clarify more refined biological links between periodontitis and diabetes-related genitourinary complications from multiple perspectives, including oral microbial dysbiosis, host immune response, and metabolic abnormalities (Yamazaki and Kamada, 2024). Future studies should also integrate host genetic susceptibility, inflammatory biomarkers, microbiome profiles, and multi-omics approaches to better identify susceptible patient subgroups and clarify the biological plausibility of this proposed axis. Furthermore, future research should also strengthen the screening of biomarkers and explore precision medicine pathways integrating clinical phenotypes, inflammatory characteristics, and microbiome data (Gundelly et al., 2024). These studies may help promote the development of risk stratification and individualized management strategies for patients with diabetes and periodontitis, while further determining whether periodontal inflammation has clinically meaningful relevance to diabetes-related genitourinary outcomes.
